# Supplementary material for: Less is more: Qualitative insights into physically active learning in secondary math education
Source: PLoS One. 2025 Nov 18;20(11):e0336641. doi: 10.1371/journal.pone.0336641 (PMC12626303; doi:10.1371/journal.pone.0336641)
Supplement: Attachment — Submitted filename: Response to Reviewers.docx [file pone.0336641.s002.docx]

Dear Editor Ph.D. Francesca D’Elia,

Thank you for reviewing our manuscript and for providing us with the reviewers' comments. We are very grateful for the time and effort you and the other reviewers have devoted to evaluating our work.

We understand and recognize the importance of the concerns raised in improving the quality of the manuscript. We have carefully considered all the comments and hope that the revised version addresses each of your concerns thoroughly. We are optimistic that these revisions will strengthen the manuscript and align it with the standards of *Plos One.*

You will find a point-by-point response to the comments below, starting with yours and continuing with those of each reviewer. All changes to the original manuscript have been highlighted using the track change’s function.

Thank you once again for the opportunity to submit a revised version of the manuscript.

Yours sincerely,

**EDITOR**

**Comment:**

Affiliation: ensure consistency in listing affiliations (e.g., some institutions are listed with full addresses, others are abbreviated)

**Answer:**

We have reviewed the list of affiliations and modified it to ensure consistency in the use of abbreviations and addresses. We would like to clarify that the name “GALENO” is not an abbreviation but the full name. On the other hand, the language is established according to the internal rules of each institution (lines 8-18):

*“^1^GALENO Research Group, Department of Physical Education, Faculty of Education Sciences, University of Cadiz, Puerto Real, Spain.*

*^2^ Instituto de Investigación e Innovación Biomédica de Cádiz (INiBICA), Cadiz, Spain.*

*^3^Faculty of Health Studies, University of Bradford, Richmond Road, Bradford, United Kingdom.*

*^4^Centre for Applied Education Research, Wolfson Centre for Applied Health Research, Bradford Teaching Hospitals National Health Service Foundation Trust, Bradford Royal Infirmary, Duckworth Lane, Bradford, United Kingdom.*

*^5^ Análisis Comportamental de la Actividad Física y el Deporte (ACAFYDE) research group, Department of Didactics of Musical, Plastic and Body Expression, Faculty of Sports Sciences, University of Extremadura, Cáceres, Spain”.*

**Comment:**

Abstract: remove redundancy (e.g. “better content retention and understanding” could be simplified to “enhanced retention”).

**Answer:**

Thank you very much for your feedback. We have removed the redundancy by replacing the case in question with the term suggested by the editor (lines 37–41):

*“Although teachers and students perceived that the amount of content covered in a PAL lesson was lower compared to traditional lessons, they emphasized that learning was more meaningful due to enhanced retention, facilitated by active engagement and dynamic teaching approaches”.*

**Comment:**

 Method Section: Table 1 Caption Revision Suggestion. The current caption for Table 1 (“Summary of the sample of participants”) does not accurately reflect the data presented. Since the table specifically details the sub-sample of students who participated in the focus groups, as well as the teachers and researchers involved, it is recommended to revise the caption for clarity. Suggested alternative: “Participants in the focus groups: sub-sample overview.”

**Answer:**

Thank you for your comment. We agree that the original title did not accurately reflect the content of the table. We have therefore updated the title to: *“Participants in the interviews and focus groups: sub-sample overview”.* We have chosen to include the word *“interviews”* in the suggested proposal to adequately represent the participation of both students and teachers, since, as the editor mentions, the sample of teachers participating in the interviews is also reflected in the table.

**Comment:**

Data analysis. Clarify whether data saturation was planned or emerged organically.

**Answer:**

Thank you for your comment. We appreciate the opportunity to clarify this point. Our intention with this study was not to achieve saturation but rather to interview all the participants who consented to be interviewed to achieve information power [1]. Typically, participants are recruited until the data reach a level of saturation (i.e., wherein no new themes are identified through further data collection [2]. However, saturation is affected by several factors including the quality of the interviews and the scope of the study. The number of interviews and focus groups was selected in advance based on the study’s scope and the research team’s logistical capacity. However, as the analysis progressed, we observed that no new themes emerged after the third teacher interview and the fifth focus group, indicating that thematic saturation had been reached. Despite this, all six focus groups and five interviews were analyzed in full to ensure the richness and diversity of participants’ perspectives was fully captured. We have clarified this information in lines (250-256) of the manuscript:

*“Although the number of interviews and focus groups was determined in advance based on the study’s scope and available resources, data saturation was identified organically during the analysis, when no new themes or categories emerged. Saturation was perceived to have been achieved after the 5th focus group and the 3rd interview, where recurring patterns and consistent themes were observed across both teachers' and students' responses. This repetition of the findings provided confidence that the data adequately captured the range of experiences and perceptions of the participants”.*

1. Malterud K, Siersma VD, Guassora AD. Sample Size in Qualitative Interview Studies: Guided by Information Power. *Qualitative Health Research*. 2015;26(13):1753-1760. [doi.org/10.1177/1049732315617](https://doi.org/10.1177/1049732315617444)
2. Fusch L & Ness, P. Are we there yet? Data saturation in qualitative research. *Qualitative Report.* 2015; 20(9), 1409-1416.

**Comment:**

 Results Section: replace phrases like “PAL helped some students to keep up with the pace of the class” with more precise language such as “PAL supported student pacing for those who typically struggle with classroom instruction.”

**Answer:**

Thank you for your comment. We have modified this information in accordance with your suggestion.

**Comment:**

 Discussion: strengthen transitions between studies cited, some paragraphs begin abruptly with literature comparisons and clarify the role novelty played in the motivation observed, currently mentioned but not critically examined.

**Answer:**

Thank you very much for your observations. We have reviewed the discussion section and made changes across it to ensure greater consistency and coherence compared with the existing literature.

Additionally, we have expanded the information regarding the potential role of novelty in the observed motivation, as suggested by the editor (lines 438–444):

*“However, it is important to consider whether the motivational benefits of PAL may be partially linked to its novel approach, as the satisfaction of the need for novelty seems to predict intrinsic motivation, as well as students' participation and intention to engage in physical activity [37,38]. As in physical education, this suggests that introducing new and varied proposals, such as PALs, can increase motivation precisely because they satisfy this basic psychological need, and therefore, if overused, its impact on engagement and learning could diminish over time”.*

**Comment:**

 Conclusion: rephrase “Taken together, the findings…” to something sharper like “Collectively, the findings underscore…”

**Answer:**

Thank you for your feedback. We have rephrased this statement in accordance with your suggestion.

**Comments:**

Formatting & Language:
- Fix small typos: e.g., “seasonable” in Research data availability should likely be “reasonable.”
- Ensure consistent formatting in references: use either “Available at” or DOI, not both inconsistently.

**Answer:**

Thank you very much for your comments. We have corrected the error identified by the editor and thoroughly reviewed and amended the list of references to ensure consistent formatting.

**REVIEWER 1**

**Comment:**

I consider it to be a rigorous and high-quality work. It is true that some of the articles published by you have very similar results to those presented in this paper (Example: A mixed-methods approach of the effect of physically active learning on time-on-1 task in the secondary education class: the ACTIVE CLASS study). Understanding that an exhaustive bibliographic review has been carried out and is thus contemplated in the references section, however there is a study from Finland very similar to yours that it would be advisable to include:
Sirpa Sneck, Heidi Syväoja, Sanna Järvelä & Tuija Tammelin (2023) More active lessons: teachers’ perceptions of student engagement during physically active maths lessons in Finland, Education Inquiry, 14:4, 458-479, DOI:10.1080/20004508.2022.2058166

**Answer:**

Thank you very much for your kind comments and for taking the time to review our manuscript. As you point out, our previously published article *“A mixed-methods approach to the effect of physically active learning on time-on-task in secondary education classes: the ACTIVE CLASS study”,* presents qualitative results derived from teacher interviews. However, we believe that, while there are similarities in areas such as student behavior and participation, the present study makes a substantially different contribution. Specifically, it provides a broader and deeper insight into the implementation of PAL by integrating the perceptions of both teachers and participating students. Unlike the aforementioned study, which exclusively extracted teachers' perceptions related to time-on-task, this study covers additional aspects such as motivation, depth of learning, social interaction and the feasibility of the intervention. As the reviewer understands, this study is cited in the references section.

Regarding the study by Sneck et al. (2023) that was suggested by the reviewer, as the reviewer himself points out, it is an article similar to the present research, with the peculiarities that it analyses only the perspectives of teachers and not those of students, and that it is developed in primary education, unlike our research, which is developed in secondary education. This study was cited in our manuscript as reference 25. However, we have reviewed the information in our manuscript regarding this study and expanded upon it in lines (93–102):

*“This qualitative perspective has been addressed by several studies in primary education, which focus separately on teachers' perceptions [25,26], students' perceptions [27] and some that combine both perspectives [28,29]. The main findings of these studies indicate an increase in students' engagement and concentration during PAL lessons [25]. They also consider PAL as a method that introduces variation and enjoyment into learning [25,28,29], improves social relationships between peers and promoting quality learning [25,26,29]. However, some teachers have difficulty identifying its cognitive and social benefits when applying it to students with cognitive challenges due to their support needs. Furthermore, although they report that it is another path to learning, they are uncertain about achieving learning objectives [25]”.*

**Comment:**

In another order of analysis of your article, you have been able to observe that in line 115, they set up a chair saying: "In addition, no study to date included perspectives of students and teachers together" which is not entirely true, considering that in the journal PLOS ONE, there is an article with that double approach that you refer to as an essential value of your studies. I therefore recommend that you review this article and take into account the reformulation of some of its sentences.
Simard L, Bouchard J, Lavallière M, Chevrette T. Promoting physical activity and academic achievement through physically active learning: Qualitative perspectives of co-design and implementation processes. PLoS One. 2023 Nov 22;18(11):e0294422. doi: 10.1371/journal.pone.0294422. PMID: 37992080; PMCID: PMC10664963

**Answer:**

Thank you for your comment. As the reviewer rightly points out, there are already studies that evaluate the perceptions of both teachers and students of PAL. However, these studies have focused on primary education. In the highlighted sentence, we intended to refer to the context of secondary education, as, to our knowledge, no studies have yet been conducted that include both perspectives in this context. We understand that this sentence may be confusing, so we have made minor changes to the sentence to clarify this (lines 116–118):

*“Despite the emerging research on PAL in secondary education, an important gap in understanding its impact and implementation remains. Furthermore, no study to date has included both students and teacher perspectives together in secondary education”.*

**REVIEWER 2**

**Comment:**

The manuscript addresses a highly relevant and important topic. The article explores Physically Active Learning (PAL), a pedagogical approach that integrates physical activity into core subject lessons to combine physical and academic benefits. Despite its potential, implementing PAL in secondary education remains challenging, making it crucial to understand teachers' and students' perceptions and experiences. The study's primary aim was to assess the perceptions and experiences of teachers and students after participating in a PAL intervention in secondary education. From the point of view of methodological rigor, the problem of the study was identified, referring its objectives, and presented scientific evidence on the subject of the study. The research variables are defined and the procedures and instruments for the research should more well explained. The results are presented, being complemented with a discussion. The conclusions are very enlightening and the references are adjusted to the topic under discussion.

**Answer:**

Thank you very much for your comments and for the time and effort you have put into reviewing our manuscript. We have considered and reviewed each of your suggestions. Below, we respond to each of them individually.

**Comment:**

“Setting and participants” line 148: the number of participants in the study are not clear. The authors written “ …A total of 104 students 148 (aged 12-14 years, n= 45 boys) and five mathematics teachers (two male) were included in the study…” what happened to the others 103 students? And were all from the same gender?

**Answer:**

Thank you for your feedback. We would like to clarify that, as mentioned in the design section, this particular study emerged from the ACTIVE CLASS project, in which the group that implemented the physically active learning intervention consisted of a total of 104 students. Of those 104 participants, this qualitative study, due to its nature, included a subsample of 36 students (n= 18 boys), as it could not cover the entire sample of the intervention group. In order to avoid confusion, we have slightly modified this information in lines (149-159):

*“Participant recruitment took place from November 2022 to January 2023. The study was explained to the participants before starting, and the volunteers, parents, or tutors provided written informed consent. A total of 104 students (aged 12-14 years, n= 45 boys) and five mathematics teachers (two male) participated in the PAL intervention. To be included in the analysis, all teachers involved in the intervention were invited to participate in the interviews. On the other hand, from the total number of students, a sub-sample of 36 (six students per participating class [n= 18 boys]) were invited to participate and completed the focus groups for data collection (table 1). This sub-sample of students was randomly selected within each participating class, with a balanced gender distribution to ensure homogeneity in the composition of the group. Neither students nor teachers had been exposed to previous PAL experiences”.*

Additionally, we have modified the title of Table 1 to: *"**Participants in the interviews and focus group: sub-sample overview”.*

**Comment:**

“Setting and participants” line 159, table 1: why is important to include the information about the two researchers? Why there is a frequency of each student? Why the authors don´t present the mean and SD of each variable concerning participants (i.e..: Age, sex. School year)? Concerning teachers the authors should explain better the meaning of a teacher belong a some class, what means class 1, class 4 and 5…

**Answer:**

Thank you for your comment. We considered it relevant to include information about the two researchers because, as explained in: *“Consolidated criteria for reporting qualitative research (COREQ): a 32-item checklist for interviews and focus groups”* [1], qualitative researchers are closely involved in the research process and with the participants, which makes it difficult to completely avoid personal bias. Therefore, researchers should acknowledge and clarify to readers their identity, credentials, occupation, gender, experience, and training. This enhances the credibility of the results, as it allows readers to assess how these factors may have influenced the researchers' observations and interpretations.

1. Tong A., Sainsbury P., & Craig J. Consolidated criteria for reporting qualitative research (COREQ): a 32-item checklist for interviews and focus groups. *International journal for quality in health care*. 2007; *19*(6): 349-357. [doi.org/10.1093/intqhc/mzm042](https://doi.org/10.1093/intqhc/mzm042)

On the other hand, with regard to the students, we have decided to show the characteristics of each one in the table rather than the average, as the sample is relatively small. This allows us to demonstrate the diversity of profiles more accurately, helping the reader to understand the distribution of the focus groups and contextualize the textual quotations included in the results. They can easily identify the specific characteristics (gender, age and class group) of the student who provided each testimony.

With regard to teachers, we have included information in the table legend to clarify what it means for a teacher to correspond to each of the classes (lines 163-164):
*“Note. The class section refers to the specific class group where each teacher taught their lessons. In the case of students, this same number was also used to differentiate the focus groups”.*

**Comment:**

Should be explain some data about the control group, how many students, age, sex, school year…

**Answer:**

Thank you for your comment. As we explained to the reviewer in previous comments, this study emerged from the ACTIVE CLASS project, which was a randomized controlled trial involving two experimental groups and one control group. For this specific qualitative study, however, we only included the experimental group. This information can be found in (lines 130–136):

*“This study is part of the ACTIVE CLASS randomized controlled trial (registration number: NCT05891054). The ACTIVE CLASS study was conducted simultaneously in the Spanish provinces of Cadiz and Caceres and included a total of two experimental groups (Active Break intervention group and PAL intervention group) and one control group. More information regarding the study protocol is available elsewhere [33]. The present study included only the PAL intervention group, which completed a 16-week intervention based on including PAL lessons in the subject of mathematics”.*

The specific objective of this study was to explore in depth the perceptions and experiences of teachers and students who actively participated in the PAL intervention. Given that the control group was not exposed to this intervention and did not make any changes to their usual teaching methodology, we considered that it was not relevant to include their characteristics in this qualitative analysis, which focused exclusively on the participants in the intervention.

Furthermore, as we reported in your previous comments, we have modified the information in the “*settings and participants”* section to avoid confusion in this regard.

**Comment:**

It´s important to explain how many schools were involved in the program.

**Answer:**

Thank you for your comment. We report this information in lines (142-145):

*“Of the six public secondary schools included in the ACTIVE CLASS study, a randomization process was carried out and two schools were selected, which were assigned to the PAL intervention group and included in the present study”.*

**Comment:**

“Intervention” lines 164, 165 – the PAL program should be more well explained. For example: How many times per week and minutes was the program? Concerning the physical conditions variables, where they all integrate in the program. The experimental group how many traditional (in room) math class have and how many have in outdoor space per week? And the control group?

**Answer:**

Thank you for your observations. We have restructured the section, in order to incorporate the information requested by the reviewer as well as to provide clearer information about how the developed intervention was implemented (lines 166-194):

*“The intervention consisted of including physical activity in mathematics lessons without modifying the content of the subject for 16 weeks. For this purpose, a weekly PAL lesson, each lasting 55 minutes, was performed outside the classroom, assuming a total of 16 PAL lessons, all of which were conducted outdoors. In these lessons, activities such as moving, jumping, throwing, etc. were integrated into the activities of the mathematics subject. To ensure consistency across all PAL lessons, they were always conducted on the same day of the week and at the same time during the 16-week intervention period.*

*Each PAL lesson began with the teacher introducing the educational content to be covered in the lesson (i.e. equations, geometry, fractions...). The activities developed in the lesson consisted of incorporating the students' movement into the mathematical exercises normally found in the textbook (i.e. proportionality problems, addition and subtraction of decimal numbers...) through games, collaborative tasks or competitive activities. Specific examples of activities that follow our PAL methodology can be found here. This resource was made available to teachers to support them in implementing PAL lessons during the intervention and to provide them with tools to further develop this pedagogical approach in the future, if deemed appropriate.*

*The mathematics teacher was responsible for sharing the PAL lessons thought the intervention. However, a specialized technician was present at each session to provide practical support to the teachers and to ensure the correct development of the activities. In addition, lessons were co-designed by the mathematics teachers and members of the research team who had expertise in integrating physical activity into educational settings. Thus, prior to the development of each PAL lesson, a meeting was held between the research team and each mathematics teacher. In these meetings, the curricular content that the teacher wanted to teach in the lesson was discussed and the activities to incorporate physical activity into these lessons. Although the researchers aimed to incorporate physical activity to promote student engagement and potential health benefits, the primary focus remained on ensuring the effective delivery of the mathematics content and maintaining alignment with curriculum objectives. This approach sought to balance the dual benefits of promoting learning and increasing physical activity in a structured way”.*

**Comment:**

“Data Collection” lines 204, 205 – the question of the interview is repeated.

**Answer:**

Thank you for your appreciation, we have removed this repetition.

**Comment:**

The development and validation process of the instruments-interviews (students and teachers) should be more well explained.

**Answer:**

Thank you very much for your comment. We have taken your suggestion into account and introduced changes in the “data collection” section in order to provide more explanation about the development and validation of the instruments used (lines 196-219):

*“Semi-structured interviews with teachers (n= 5) and student focus groups (n= 6) were used as techniques for qualitative data collection. Both were conducted after the end of the intervention period of the study (June 2023). These methods are supported in educational research for their ability to provide rich, contextual data [34,35].*

*The research team collaborated in the development of the interview and focus group guides. A first draft of the questions was developed based on previous PAL research and the team's own experience in implementing and evaluating similar interventions. This draft was then revised and refined through multiple rounds of discussion among all authors, including researchers with expertise in qualitative methods, allowing the team to assess the relevance, clarity and completeness of the questions. In both cases, the questions were focused on describing their experiences with PAL.*

*Some examples of questions included were: “What do you think about the pedagogical approach of PAL?”, “what are the benefits you think PAL has brought you?”, “what disadvantages or barriers do you find in using PAL?”, “what differences do you find in terms of work and learning content compared to a traditional lesson?”, “how do you think PAL is different from a traditional lesson?”.*

*In addition, participants were encouraged to express their opinions and experiences on themes beyond the initial guide, promoting the emergence of unanticipated but relevant topics.*

*The interviews and focus groups on each school were conducted by the same researcher to promote trust and familiarity. Both were conducted in the mathematics department to provide a pleasant and quite environment for the participants. In both cases, the interviews lasted between 20-40 minutes (with an average of 30.6 min) and were audio recorded for later transcription”.*

**Comment:**

Could be interesting to understand if the academic results were similar between the students that integrate the experimental and control groups.

**Answer:**

Thank you very much for your comment. We agree that it would be interesting to explore whether academic outcomes differ between students in experimental and control groups. This is certainly a relevant line of analysis that could be addressed in future quantitative articles derived from this study. However, as mentioned in a previous response, we did not consider collecting qualitative data from the control group in this particular study, as the specific aim was to explore in depth the perceptions and experiences of participants who engaged directly with the PAL intervention.

**Comment:**

The chapter “Limitations and strengths” it is very long it is suggested to be shorter.

**Answer:**

Thank you for your suggestion. We have taken it into account and reduced this section (lines 540-569):

*“The present study is not without some limitations. Firstly, for logistical reasons, the focus groups were conducted with a randomized sub-sample of students. This may affect the representativeness of the results, as the inclusion of the total sample could have provided a completer and more diverse picture of their perceptions and experiences. Although the authors feel that they have reached saturation in terms of data generation, the results should not be generalized to all Spanish schools. Nevertheless, the consistency of themes across both participating schools suggests potential transferability. Another limitation is the support provided by the research team during the intervention, which, although valuable in ensuring fidelity, may have influenced teachers' experiences by facilitating the implementation of the approach, as well as limiting its scalability. Implementation of PAL on a larger scale would require investment in teacher training and provision of resources. Finally, the study highlights the challenge of addressing different learning paces in PAL classes. Students who struggle with the subject may find it more difficult to engage, which could impact both individual learning and group dynamics. To ensure inclusive implementation, as well as the viability and sustainability of PAL, adaptable and sufficient teacher support strategies and resources are needed.*

*However, the study also has several significant strengths that add value to the research. Firstly, it is the first study in secondary education to assess both teachers' and students' perceptions of PAL, providing a valuable contrast of perspectives and a more complete picture of the implementation and impact of the intervention. In addition, the sample included a heterogeneous range of teachers with different profiles and ages, as well as students randomly selected from different classes, with an equal gender distribution. This selection process not only enriched the diversity of perspectives on PAL lessons but also facilitated the inclusion of students with different levels of engagement, allowing for a fuller understanding of different views and experiences. None of the participants had previous experience of PAL, which provided a fresh and unbiased perspective. Finally, the interviews were conducted by the same people in each school who had been present throughout the intervention. This not only made participants feel more comfortable in expressing their experiences, but also ensured consistency in data collection, thereby increasing the reliability of the findings”.*

**Comment:**

The manuscript should respect the structure defined by Plos One guidelines.

**Answer:**

Thanks for your comments, we have reviewed the sample formats provided by Plos One and adapted the structure of the manuscript to this format.
